# Supplementary material for: Clostridioides difficile colonization amplification despite limited in-hospital transmission: A modeling study
Source: PLoS Med. 2026 Apr 13;23(4):e1004712. doi: 10.1371/journal.pmed.1004712 (PMC13120704; doi:10.1371/journal.pmed.1004712)
Supplement: S1 Fig — Scatter plot showing the relationship between Ri and Ai across 1,000 simulations with randomly sampled parameter values. Each point represents the outcome from a single parameter set. (DOCX) [file pmed.1004712.s001.docx]

**S1 Figure.** Relationship between intrinsic reproduction number and colonization amplification index.

**
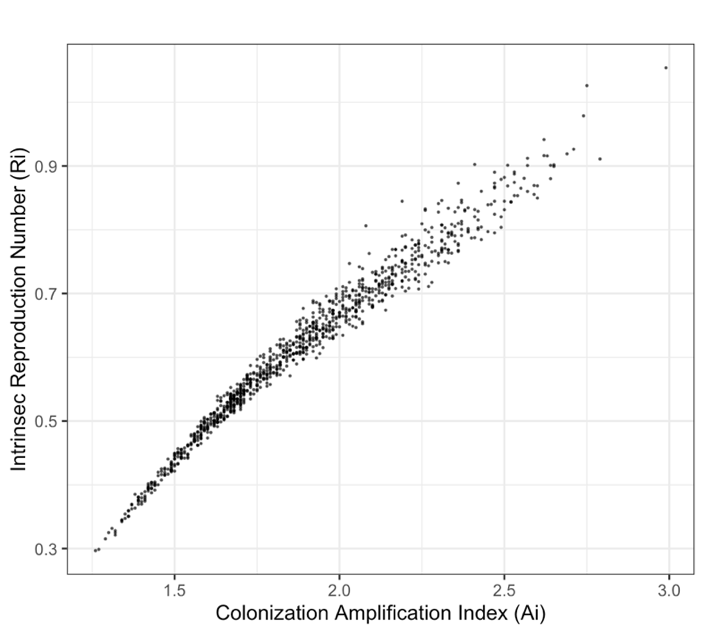
**

Scatter plot showing the relationship between Ri and Ai across 1,000 simulations with randomly sampled parameter values. Each point represents the outcome from a single parameter set.
