# Supplementary material for: Clostridioides difficile colonization amplification despite limited in-hospital transmission: A modeling study
Source: PLoS Med. 2026 Apr 13;23(4):e1004712. doi: 10.1371/journal.pmed.1004712 (PMC13120704; doi:10.1371/journal.pmed.1004712)
Supplement: S1 Table — Partial rank correlation coefficients (PRCC) were obtained after Monte Carlo sampling of parameter values. The PRCC provides adjusted correlation values between model parameters and the intrinsic reproduction number. A cutoff of 3 days was used to distinguish community-associated (CA-CDI) from healthcare-associated CDI (HCA-CDI). (DOCX) [file pmed.1004712.s002.docx]

**S1 Table.** Partial rank correlation coefficients for input parameters in the intrinsic reproduction number estimates.

| **Symbol** | **Parameter** | **PRCC** | **Lower** | **Upper** |
| --- | --- | --- | --- | --- |
| δ | Transmission rate of infected patients | 0.936 | 0.914 | 0.958 |
| x | Relative transmissibility for colonized patients | 0.811 | 0.775 | 0.848 |
| α | Rate of antibiotic use | 0.495 | 0.441 | 0.549 |
| z | Fraction of susceptible patients admitted | 0.485 | 0.43 | 0.539 |
| $\Psi$_4_ | Discharge rate of symptomatic patients | 0.012 | -0.05 | 0.074 |
| h_1_ | Bacterial clearance rate due to treatment for infected patients | -0.002 | -0.065 | 0.06 |
| σ_1_ | Effective fraction of asymptomatic carriers clearing colonization following treatment | -0.087 | -0.148 | -0.025 |
| ɣ_1_ | Reduction constant of transmission due to contact precautions in infected individuals | -0.117 | -0.179 | -0.055 |
| f_1_ | Diagnosis rate of infected patients | -0.129 | -0.190 | -0.067 |
| ν | Progression rate to symptomatic disease | -0.139 | -0.200 | -0.077 |
| ε | Fraction of asymptomatic carriers who develop symptomatic disease | -0.192 | -0.253 | -0.131 |
| $\Psi$_1_ | Discharge rate of non-susceptible patients | -0.496 | -0.549 | -0.442 |
| $\Psi$_3_ | Discharge rate of asymptomatic carriers | -0.746 | -0.788 | -0.705 |
| $\Psi$_2_ | Discharge rate of susceptible patients | -0.83 | -0.865 | -0.795 |

Partial rank correlation coefficients (PRCC) were obtained after Monte Carlo sampling of parameter values. The PRCC provides adjusted correlation values between model parameters and the intrinsic reproduction number. A cutoff of three days was used to distinguish community-associated (CA-CDI) from healthcare-associated CDI (HCA-CDI).
