# Supplementary material for: Clostridioides difficile colonization amplification despite limited in-hospital transmission: A modeling study
Source: PLoS Med. 2026 Apr 13;23(4):e1004712. doi: 10.1371/journal.pmed.1004712 (PMC13120704; doi:10.1371/journal.pmed.1004712)
Supplement: S2 Table — Partial rank correlation coefficients (PRCC) for the colonization amplification index were obtained after Monte Carlo sampling of parameter values. A cutoff of 3 days was used to distinguish CA-CDI from HCA-CDI. (DOCX) [file pmed.1004712.s003.docx]

**S2 Table.** Partial rank correlation coefficients for input parameters in the colonization amplification index estimates.

| **Symbol** | **Parameter** | **PRCC** | **Lower** | **Upper** |
| --- | --- | --- | --- | --- |
| δ | Transmission rate of infected patients | 0.928 | 0.905 | 0.951 |
| x | Relative transmissibility for colonized patients | 0.78 | 0.741 | 0.819 |
| z | Fraction of susceptible patients admitted | 0.576 | 0.525 | 0.627 |
| α | Rate of antibiotic use | 0.533 | 0.48 | 0.585 |
| h_1_ | Bacterial clearance rate due to treatment for infected patients | 0.012 | -0.05 | 0.074 |
| ν | Progression rate to symptomatic disease | -0.001 | -0.063 | 0.061 |
| ε | Fraction of asymptomatic carriers who develop symptomatic disease | -0.025 | -0.087 | 0.037 |
| f_1_ | Diagnosis rate of infected patients | -0.049 | -0.111 | 0.013 |
| $\Psi$_4_ | Discharge rate of symptomatic patients | -0.117 | -0.179 | -0.055 |
| σ_1_ | Effective fraction of asymptomatic carriers clearing colonization following treatment | -0.14 | -0.202 | -0.079 |
| ɣ_1_ | Reduction constant of transmission due to contact precautions in infected individuals | -0.145 | -0.207 | -0.084 |
| $\Psi$_1_ | Discharge rate of non-susceptible patients | -0.52 | -0.573 | -0.467 |
| $\Psi$_3_ | Discharge rate of asymptomatic carriers | -0.744 | -0.786 | -0.703 |
| $\Psi$_2_ | Discharge rate of susceptible patients | -0.814 | -0.850 | -0.778 |

Partial rank correlation coefficients (PRCC) for the colonization amplification index were obtained after Monte Carlo sampling of parameter values. A cutoff of three days was used to distinguish community-associated (CA-CDI) from healthcare-associated CDI (HCA-CDI).
