# Supplementary material for: Clostridioides difficile colonization amplification despite limited in-hospital transmission: A modeling study
Source: PLoS Med. 2026 Apr 13;23(4):e1004712. doi: 10.1371/journal.pmed.1004712 (PMC13120704; doi:10.1371/journal.pmed.1004712)
Supplement: S3 Table — Each parameter was varied individually across a predefined range while all other parameters were held at their baseline values. The resulting values of the intrinsic reproduction number and the colonization amplification index are shown for each parameter value. (DOCX) [file pmed.1004712.s004.docx]

**S3 Table.** Univariate sensitivity analysis of model parameters and their impact on the intrinsic reproduction number and the colonization amplification index.

| **Parameter**  **(units)** | **Parameter  value** | **Intrinsic  Reproduction  Number** | **Colonization  Amplification  Index** | **Parameter** | **Parameter  value** | **Intrinsic  Reproduction  Number** | **Colonization  Amplification  Index** |
| --- | --- | --- | --- | --- | --- | --- | --- |
| ε: Fraction of asymptomatic carriers developing symptoms during hospitalization (dimensionless) | 0.040 | 1.90 | 3.98 | n: Overall proportion of  asymptomatic carriers  at admission  (dimensionless) | 0.020 | 1.22 | 7.25 |
|  | 0.050 | 1.39 | 3.37 |  | 0.030 | 1.11 | 4.84 |
|  | 0.060 | 1.14 | 2.96 |  | 0.040 | 1.00 | 3.64 |
|  | 0.070 | 0.99 | 2.67 |  | 0.050 | 0.89 | 2.92 |
|  | 0.090 | 0.80 | 2.27 |  | 0.060 | 0.79 | 2.44 |
|  | 0.110 | 0.69 | 2.02 |  | 0.070 | 0.70 | 2.10 |
|  | 0.130 | 0.61 | 1.85 |  | 0.080 | 0.61 | 1.85 |
|  | 0.150 | 0.54 | 1.72 |  | 0.090 | 0.52 | 1.65 |
|  | 0.170 | 0.50 | 1.62 |  | 0.100 | 0.44 | 1.49 |
|  | 0.190 | 0.46 | 1.54 |  | 0.110 | 0.36 | 1.36 |
|  | 0.210 | 0.42 | 1.48 |  | 0.120 | 0.28 | 1.25 |
|  | 0.230 | 0.39 | 1.43 |  | 0.130 | 0.21 | 1.16 |
| f_1_: Diagnosis rate of  infected patients (Days^-1^) | 0.500 | 0.65 | 1.94 | σ_1_: Effective fraction of asymptomatic carriers clearing colonization following treatment (dimensionless) | 0.45 | 0.61 | 1.87 |
|  | 0.526 | 0.64 | 1.92 |  | 0.50 | 0.61 | 1.87 |
|  | 0.556 | 0.63 | 1.90 |  | 0.55 | 0.61 | 1.86 |
|  | 0.588 | 0.63 | 1.88 |  | 0.60 | 0.61 | 1.86 |
|  | 0.625 | 0.62 | 1.86 |  | 0.65 | 0.61 | 1.85 |
|  | 0.667 | 0.61 | 1.85 |  | 0.70 | 0.61 | 1.85 |
|  | 0.714 | 0.60 | 1.83 |  | 0.75 | 0.61 | 1.84 |
|  | 0.769 | 0.59 | 1.81 |  | 0.80 | 0.61 | 1.84 |
|  | 0.833 | 0.58 | 1.79 |  | 0.85 | 0.61 | 1.83 |
|  | 0.909 | 0.57 | 1.77 |  | 0.90 | 0.61 | 1.83 |
|  | 1.000 | 0.56 | 1.75 |  | 0.95 | 0.60 | 1.82 |
| ɣ_1_: Reduction of transmission due to contact precautions in infected individuals (dimensionless) | 0.450 | 0.61 | 1.85 | v: Progression rate to symptomatic disease  (Days^-1^) | 0.091 | 1.26 | 3.17 |
|  | 0.500 | 0.61 | 1.85 |  | 0.100 | 1.16 | 2.98 |
|  | 0.550 | 0.61 | 1.85 |  | 0.111 | 1.05 | 2.79 |
|  | 0.600 | 0.61 | 1.85 |  | 0.125 | 0.96 | 2.61 |
|  | 0.650 | 0.61 | 1.85 |  | 0.143 | 0.87 | 2.42 |
|  | 0.700 | 0.61 | 1.85 |  | 0.167 | 0.78 | 2.23 |
|  | 0.750 | 0.61 | 1.85 |  | 0.200 | 0.70 | 2.04 |
|  | 0.800 | 0.61 | 1.85 |  | 0.250 | 0.61 | 1.85 |
|  | 0.850 | 0.61 | 1.85 |  | 0.333 | 0.51 | 1.66 |
|  | 0.900 | 0.61 | 1.85 |  | 0.500 | 0.41 | 1.46 |
|  | 0.950 | 0.61 | 1.85 |  | 1.000 | 0.30 | 1.27 |
| h_1_: Bacterial clearance rate due to treatment for infected patients or treatment duration (Days^-1^) | 0.067 | 0.61 | 1.84 | x: Relative transmissibility  for colonized patients  (dimensionless) | 0.45 | 0.60 | 1.85 |
|  | 0.071 | 0.61 | 1.84 |  | 0.50 | 0.60 | 1.85 |
|  | 0.077 | 0.61 | 1.84 |  | 0.55 | 0.60 | 1.85 |
|  | 0.083 | 0.61 | 1.84 |  | 0.60 | 0.60 | 1.85 |
|  | 0.091 | 0.61 | 1.84 |  | 0.65 | 0.61 | 1.85 |
|  | 0.100 | 0.61 | 1.85 |  | 0.70 | 0.61 | 1.85 |
|  | 0.111 | 0.61 | 1.85 |  | 0.75 | 0.61 | 1.85 |
|  | 0.125 | 0.61 | 1.85 |  | 0.80 | 0.61 | 1.85 |
|  | 0.143 | 0.61 | 1.85 |  | 0.85 | 0.61 | 1.85 |
|  | 0.167 | 0.61 | 1.85 |  | 0.90 | 0.61 | 1.85 |
|  | 0.200 | 0.61 | 1.85 |  | 0.95 | 0.61 | 1.85 |
| m: Fraction of admitted asymptomatic carriers to the E compartment  (dimensionless) | 0.070 | 0.79 | 2.23 | z: Fraction of susceptible  patients admitted  (dimensionless) | 0.02 | 0.70 | 1.84 |
|  | 0.080 | 0.75 | 2.16 |  | 0.06 | 0.67 | 1.84 |
|  | 0.090 | 0.71 | 2.08 |  | 0.10 | 0.65 | 1.84 |
|  | 0.100 | 0.68 | 2.00 |  | 0.14 | 0.63 | 1.84 |
|  | 0.110 | 0.64 | 1.92 |  | 0.18 | 0.62 | 1.84 |
|  | 0.120 | 0.61 | 1.85 |  | 0.22 | 0.61 | 1.85 |
|  | 0.130 | 0.57 | 1.77 |  | 0.26 | 0.60 | 1.85 |
|  | 0.140 | 0.53 | 1.69 |  | 0.30 | 0.59 | 1.85 |
|  | 0.150 | 0.49 | 1.61 |  | 0.34 | 0.58 | 1.85 |
|  | 0.160 | 0.45 | 1.53 |  | 0.38 | 0.57 | 1.85 |
|  | 0.170 | 0.41 | 1.46 |  | 0.42 | 0.57 | 1.85 |
| α: Rate of antibiotic use (Days^-1^) | 0.051 | 0.61 | 1.84 | Ψ_1_: Discharge rate of non-susceptible patients (Days^-1^) | 0.133 | 0.60 | 1.85 |
|  | 0.054 | 0.61 | 1.84 |  | 0.140 | 0.60 | 1.85 |
|  | 0.056 | 0.61 | 1.85 |  | 0.147 | 0.60 | 1.85 |
|  | 0.059 | 0.61 | 1.85 |  | 0.153 | 0.60 | 1.85 |
|  | 0.061 | 0.61 | 1.85 |  | 0.160 | 0.61 | 1.85 |
|  | 0.064 | 0.61 | 1.85 |  | 0.167 | 0.61 | 1.85 |
|  | 0.067 | 0.61 | 1.85 |  | 0.173 | 0.61 | 1.85 |
|  | 0.069 | 0.60 | 1.85 |  | 0.180 | 0.61 | 1.85 |
|  | 0.072 | 0.60 | 1.85 |  | 0.187 | 0.61 | 1.84 |
|  | 0.074 | 0.60 | 1.85 |  | 0.193 | 0.61 | 1.84 |
|  | 0.077 | 0.60 | 1.85 |  | 0.200 | 0.61 | 1.84 |
| λ: Admission rate (Patients/days) (dimensionless) | 60.8 | 0.91 | 2.47 | Ψ_2_: Discharge rate of susceptible patients (Days^-1^) | 0.133 | 0.61 | 1.85 |
|  | 63.8 | 0.84 | 2.32 |  | 0.140 | 0.61 | 1.85 |
|  | 66.9 | 0.77 | 2.19 |  | 0.147 | 0.61 | 1.85 |
|  | 69.9 | 0.71 | 2.06 |  | 0.153 | 0.61 | 1.85 |
|  | 73.0 | 0.66 | 1.95 |  | 0.160 | 0.61 | 1.85 |
|  | 76.0 | 0.61 | 1.85 |  | 0.167 | 0.61 | 1.85 |
|  | 79.0 | 0.56 | 1.75 |  | 0.173 | 0.61 | 1.85 |
|  | 82.1 | 0.51 | 1.66 |  | 0.180 | 0.61 | 1.85 |
|  | 85.1 | 0.46 | 1.58 |  | 0.187 | 0.61 | 1.85 |
|  | 88.2 | 0.42 | 1.50 |  | 0.193 | 0.61 | 1.85 |
|  | 91.2 | 0.38 | 1.43 |  | 0.200 | 0.61 | 1.85 |
| π: Fraction of patients admitted who have symptomatic infections (dimensionless) | 0.0040 | 0.69 | 2.00 | Ψ_3_: Discharge rate of asymptomatic carriers (Days^-1^) | 0.133 | 0.54 | 1.70 |
|  | 0.0042 | 0.67 | 1.97 |  | 0.140 | 0.55 | 1.73 |
|  | 0.0044 | 0.65 | 1.94 |  | 0.147 | 0.57 | 1.76 |
|  | 0.0046 | 0.64 | 1.91 |  | 0.153 | 0.58 | 1.79 |
|  | 0.0048 | 0.62 | 1.88 |  | 0.160 | 0.59 | 1.82 |
|  | 0.0050 | 0.61 | 1.85 |  | 0.167 | 0.61 | 1.85 |
|  | 0.0052 | 0.59 | 1.82 |  | 0.173 | 0.62 | 1.88 |
|  | 0.0054 | 0.57 | 1.79 |  | 0.180 | 0.63 | 1.90 |
|  | 0.0056 | 0.56 | 1.76 |  | 0.187 | 0.65 | 1.93 |
|  | 0.0058 | 0.54 | 1.73 |  | 0.193 | 0.66 | 1.96 |
|  | 0.0060 | 0.52 | 1.70 |  | 0.200 | 0.67 | 1.99 |
| Ψ_4_: Discharge rate of symptomatic patients (Days^-1^) | 0.067 | 0.58 | 1.79 |  | | | |
|  | 0.070 | 0.58 | 1.80 |  |  |  |  |
|  | 0.073 | 0.59 | 1.81 |  |  |  |  |
|  | 0.077 | 0.59 | 1.82 |  |  |  |  |
|  | 0.080 | 0.60 | 1.84 |  |  |  |  |
|  | 0.083 | 0.61 | 1.85 |  |  |  |  |
|  | 0.087 | 0.61 | 1.86 |  |  |  |  |
|  | 0.090 | 0.62 | 1.87 |  |  |  |  |
|  | 0.093 | 0.62 | 1.88 |  |  |  |  |
|  | 0.097 | 0.63 | 1.89 |  |  |  |  |

Each parameter was varied individually across a predefined range while all other parameters were held at their baseline values. The resulting values of the intrinsic reproduction number (Ri) and the colonization amplification index (Ai) are shown for each parameter value.
