# Supplementary material for: Clostridioides difficile colonization amplification despite limited in-hospital transmission: A modeling study
Source: PLoS Med. 2026 Apr 13;23(4):e1004712. doi: 10.1371/journal.pmed.1004712 (PMC13120704; doi:10.1371/journal.pmed.1004712)
Supplement: Attachment — Submitted filename: Responses.pdf [file pmed.1004712.s007.pdf]

Dear Editorial Team,

We sincerely thank the editorial team and the reviewers for their thoughtful feedback, which has been instrumental in improving the clarity, impact, and overall quality of our manuscript. You will find in this document a response to each of the reviewers' comments, as well as the corresponding changes highlighted in red in the updated manuscript.

Best regards,

Daniel de la Rosa Martínez, MD/PhD.
Postdoctoral Researcher
University of California San Francisco
The Francis I. Proctor Foundation
daniel.delarosamartinez@ucsf.edu

Seth Blumberg, MD/PhD.
Associate Professor
University of California San Francisco
The Francis I. Proctor Foundation
seth.blumberg@ucsf.edu

Reviewer #1:

The authors present a thought-provoking piece of modeling work based in part on real-world clinical data which calls into question some of the assumptions underpinning current C difficile prevention measures. Their modeling approach is well explained (even for a clinician and amateur modeler like me) and assumed input values well delineated (Table 1 being particularly helpful). I do have a few questions mostly related to curiosity about how the modeled results can be compared to some of the limited real world studies looking at similar numbers:

1) With the Ai of 1.9 - suggesting a 90% increase in colonization by discharge relative to admission: limited prospective surveys (such as that by Dubberke et al AAC 2015; https://journals.asm.org/doi/10.1128/aac.00642-15) do confirm increases in colonization rates between admission and discharge, but rates in this prospective microbiologic survey at least seem much lower than the 90% increase here. How might the authors reconcile this estimate with existing survey data? Is there perhaps also a time-dependent component? (e.g., rather than an overall average amplification in colonization, perhaps a per day hospitalized increase?).

Thank you for highlighting this point of confusion. As discussed below, certain characteristics of our model may affect the estimated magnitude of amplification, particularly assumptions related to the natural history of this disease and parameter uncertainty. Although we aimed to construct the best model based on available evidence and extensive sensitivity analyses, deriving a precise quantitative estimate with low uncertainty remains challenging.

With respect to the Dubberke et al study, several methodological characteristics contribute to the observed

differences (1). First, the screening method used is operator-dependent, as it relies on anaerobic culture, which is associated with a higher false-negative rate compared with molecular methods such as PCR. Second, the sample size is relatively small, with a cohort of 235 patients. Third, antibiotic exposure among patients who were not colonized at admission appears much lower compared to the medical center used for our study. In that study, high-risk antibiotics such as clindamycin, carbapenems, and fluoroquinolones were used in approximately 2%, 4%, and 12% of patients, respectively.

Our estimates appear consistent with several empirical studies reporting increases in *C. difficile* colonization with varying magnitudes. For example, Curry et al., identified that among 4,498 patients, 319 (7%) were colonized at hospital admission (2). Among those, in a subcohort of 1,432 patients with one or more follow-up tests, 124 individuals (9%) acquired asymptomatic colonization during hospitalization (2). Assuming that colonization is a long-term phenomenon, or at least persists throughout the hospital stay, the proportion of colonized individuals would therefore be higher at discharge, as reflected in our model. Similarly, among patients transferred to long-term care facilities, it has been reported that 27% of patients with at least one follow-up acquired toxigenic *C. difficile* colonization, compared with 11% observed at admission (3). Although persistence of colonization is difficult to assess due to limitations in follow-up and setting, these findings illustrate the amplification of colonization burden observed across healthcare transitions.

Regardless of the precise quantitative estimate for a specific healthcare facility, our findings demonstrate that for a broad range of reasonable parameters colonization is amplified during hospitalization. We believe this phenomena remains insufficiently considered in current surveillance and infection control strategies. Meanwhile, we acknowledge the limitations of our quantitative estimation in the main manuscript.

> Line 450: Our findings align with and complement previous reports documenting increased colonization and higher risk of CDI among individuals recently exposed to healthcare settings [18,26,28–30]. Our estimates of A_i are sensitive to parameter uncertainty, center-specific characteristics and stochastic fluctuations. These results should therefore be interpreted as a mean-field estimate, highlighting the important role of unobserved hospitalized colonized patients for the overall *C. difficile* burden in both the hospital and community settings.

1) Dubberke ER, Reske KA, Seiler S, Hink T, Kwon JH, Burnham CA. Risk Factors for Acquisition and Loss of Clostridium difficile Colonization in Hospitalized Patients. Antimicrob Agents Chemother. 2015 Aug;59(8):4533-43. doi: 10.1128/AAC.00642-15. Epub 2015 May 18. PMID: 25987626; PMCID: PMC4505269.

2) Curry SR, Hecker MT, O'Hagan J, Kutty PK, Alhmidi H, Ng-Wong YK, Cadnum JL, Jencson AL, Gonzalez-Orta M, Saldana C, Wilson BM, Donskey CJ. Natural History of Clostridioides difficile Colonization and Infection Following New Acquisition of Carriage in Healthcare Settings: A Prospective Cohort Study. Clin Infect Dis. 2023 Jul 5;77(1):77-83. doi: 10.1093/cid/ciad142. PMID: 36905149.

3) Ponnada S, Guerrero DM, Jury LA, Nerandzic MM, Cadnum JL, Alam MJ, Donskey CJ. Acquisition of Clostridium difficile Colonization and Infection After Transfer From a Veterans Affairs Hospital to an Affiliated Long-Term Care Facility. Infect Control Hosp Epidemiol. 2017 Sep;38(9):1070-1076. doi: 10.1017/ice.2017.140. Epub 2017 Jul 11. PMID: 28693636.

2) Aside from suggesting that studies of isolation or prophylactic treatment of asymptomatically colonized patients, might the authors suggest any other prospective epidemiologic studies which might provide real empirical estimates to inform model relevance/accuracy?

Thanks for suggesting this additional contextualization. Additional prospective epidemiologic studies that would strengthen empirical parameter estimation include longitudinal cohorts with repeated microbiologic testing and individual-level clinical data, including antibiotic exposure and other key determinants. Such studies would improve characterization of the natural history of colonization and enable estimation of acquisition, persistence, clearance, and other transitions between disease states. In addition, genomic sequencing–based studies linking

asymptomatically colonized individuals, symptomatic cases, and environmental isolates could quantify the contribution of colonized individuals to transmission by reconstructing transmission pathways. Finally, expanded prospective screening of healthcare workers and close contacts, including household members, could help better characterize colonization burden and reservoirs beyond hospitalized patients.

We added this in the discussion section:

> Line 517: Prospective studies with repeated testing, genomic sequencing, and expanded screening of healthcare workers could substantially improve parameter estimates and characterization of infection dynamics. In addition, these types of studies can improve model validation as model predictions can be tested prospectively.

3) Besides just the hospital component, are the authors able to account for potential roles for nursing facilities as these can serve as a source of admission, a site for discharge, and may be associated with high carriage rates as well? OK if unable - just curious.

Our in-hospital framework could incorporate varying degrees of community contribution, including admissions from other hospitals or long-term care facilities. This could be achieved by adding additional compartments or modifying admission rates of colonized or symptomatic patients to reflect heterogeneity in community case importation, which may be higher when patients are admitted from other healthcare facilities. In addition, our model can be applied to other settings, including nursing or long-term care facilities, with relatively minor modifications, including adjustment of discharge rates given the lower patient turnover compared with acute-care hospitals and the proportion of admitted colonized individuals.

> Line 523: Our model structure captures fundamental and generalizable epidemiological processes underlying *C. difficile* transmission that are applicable to other healthcare settings and can be adapted with relatively minor parameter changes or compartmental extensions under parsimonious scenarios that preserve interpretability.

Reviewer #2: This manuscript uses an epidemiological model to explore the dynamics of C. diff transmission using data from a large academic hospital. They introduce a metric to measure the amplification of host numbers between admission and exit. I am not an expert on C. diff and, as such, cannot discuss prior work in this area, so I will focus my comments on the technical aspects of the paper. This work is valuable and I am always happy to see the impact of nosocomial infection on community dynamics being investigated. In general, I think the approach applied in this work is strong, but before I can recommend publication, I had some technical questions for the authors.

222/223 - parameters were sampled from uniform distributions +/- 20% of their baseline values - You mention on lines 305/306 that some parameters are particularly poorly characterised. Given this, is a consistent choice of 20% across parameters justified, or should you widen the uncertainty bounds for some parameters and reduce them for others, conditional on the degree of uncertainty of the parameters of interest? I worry that your implied uncertainty distribution may very poorly describe the real uncertainty given this.

Thank you for helping us refine our sensitivity analysis. We agree that more attention should be given to parameters with higher uncertainty. For example, parameters representing isolation effectiveness, progression from colonization to symptomatic disease, bacterial clearance under treatment, and the relative transmissibility of asymptomatic carriers are inherently more uncertain.

In our initial submission, we addressed this limitation by conducting an extensive review of the published literature to inform parameter values and by applying a ±20% variation to reflect heterogeneity across studies. Recognizing that this range may not capture all plausible scenarios, and in response to the comment, we

performed an additional sensitivity analysis classifying parameters as high- or low-uncertainty. For high-uncertainty parameters, we expanded the plausible range, whereas for lower-uncertainty parameters, we evaluated univariate changes within the original range (S3 Table). The full sensitivity analysis is provided in the supplementary material, and the corresponding results and discussion have been added to the manuscript (S3 Table).

We observed that only three scenarios resulted in Ri> 1, and all were driven exclusively by extreme changes in ε, n, or v. The parameter ε represents the fraction of colonized individuals who develop symptoms during hospitalization; values ≤6% resulted in Ri> 1, attributable to a lower progression rate that requires a higher transmission force to reproduce the observed institutional number of symptomatic cases, leading to a higher number of acquisition events. For the parameter n, which represents the overall proportion of asymptomatic carriers at admission, values equal to or lower than 3% imply that the reduced contribution of importation is compensated by an increase in hospital-acquired cases to reach the observed number of CDI cases during the study period, thereby increasing the estimated number of secondary cases reflected in Ri. A similar mechanism explains the behavior of v: when the time from acquisition to symptom onset is prolonged (≥9 days), a higher transmission force is required to match the observed incidence.

Although the parameter combinations involving *v* and *n* are theoretically plausible, they are unlikely based on extensive existing evidence, as incubation periods are most commonly reported to be shorter than one week and colonization at admission is consistently reported above the 3% threshold (1–2). For the fraction of individuals who become colonized and subsequently develop a CDI diagnosis, evidence remains scarce; however, available cohort studies with follow-up of colonized individuals suggest values higher than 6% (3). These parameter values could correspond to outliers in our sensitivity analysis.

We added this content to the manuscript and highlight potential effects on outcomes

> Line 324: We conducted a univariate sensitivity analysis by expanding the plausible ranges of high-uncertainty parameters and performing simulations to evaluate changes in model outcomes.
>
> Line 388: In the univariate analysis, we observed similar qualitative behavior of a subcritical intrinsic reproduction number and positive colonization amplification for most scenarios. Only scenarios with low values of the fraction of colonized individuals who develop symptoms during hospitalization (ε), low colonization prevalence at admission (n), and with prolonged incubation periods (v) (≥9 days) showed R_i>1 (S4 Table). Within the explored parameter space, we observed monotonic effects on outcomes (S4 Table).
>
> Line 461: Sensitivity analyses exploring a wide range of parameters suggest that Ri < 1 for essentially all tested parameter values, with Ri > 1 observed only in a restricted set of values reflecting reduced importation of colonized patients or lower progression to symptomatic infection, conditions that require higher transmission to reproduce the observed incidence of symptomatic cases. Although such scenarios are plausible, they are less consistent with empirical observations, given that colonization at admission typically exceeds 3% and incubation periods are most commonly shorter than one week [12,17,31]. Evidence on progression from colonization to symptomatic disease is limited due to the scarcity of prospective studies with systematic follow-up; however, available data suggest that this fraction is likely greater than 6% [18].
>
> Line 38: Amplification of colonization persisted in sensitivity analyses, with R_i exceeding 1 under boundary values of low colonization prevalence at admission, a low proportion of colonized patients progressing to symptomatic disease, and prolonged incubation periods.
>
> Line 47: Our findings indicate that in most potential scenarios, in-hospital transmission of *C. difficile* is limited (R_i<1) and likely sustained by continuous importation of cases from the community.

Nevertheless, hospitalization amplifies colonization (A_i>1), which could contribute to community transmission.

1) Curry SR, Hecker MT, O'Hagan J, Kutty PK, Ng-Wong YK, Cadnum JL, Alhmidi H, Gonzalez-Orta MI, Saldana C, Wilson BM, Donskey CJ. Incubation period of *Clostridioides difficile* infection in hospitalized patients and long-term care facility residents: a prospective cohort study. Antimicrob Steward Healthc Epidemiol. 2024 Sep 24;4(1):e144. doi: 10.1017/ash.2024.392. PMID: 39346661; PMCID: PMC11428016.

2) Zacharioudakis IM, Zervou FN, Pliakos EE, Ziakas PD, Mylonakis E. Colonization with toxinogenic C. difficile upon hospital admission, and risk of infection: a systematic review and meta-analysis. Am J Gastroenterol. 2015 Mar;110(3):381-90; quiz 391. doi: 10.1038/ajg.2015.22. Epub 2015 Mar 3. PMID: 25732416.

3) Curry SR, Hecker MT, O'Hagan J, Kutty PK, Alhmidi H, Ng-Wong YK, Cadnum JL, Jencson AL, Gonzalez-Orta M, Saldana C, Wilson BM, Donskey CJ. Natural History of Clostridioides difficile Colonization and Infection Following New Acquisition of Carriage in Healthcare Settings: A Prospective Cohort Study. Clin Infect Dis. 2023 Jul 5;77(1):77-83. doi: 10.1093/cid/ciad142. PMID: 36905149.

A_i - You calculate A_i directly from the model, assuming that stability has been reached. How did you set the initial conditions for your compartments? And, if you set them semi-arbitrarily, did you check that there aren't multiple equilibria with the infection present for your models? (If there's only one, you might be able to get an analytic expression for A_i in terms of your parameters, which would be nice.)

Thanks for keeping us honest in regards to our theoretical development. We found that stability is achieved quite reliably for a range of parameter values. We also found sensitivity analyses and experiments with control did not yield odd bifurcating results suggestive of multiple equilibria. Meanwhile, we started our simulations using disease-free equilibrium, with the susceptible population obtained by solving the R and S subsystem at equilibrium. Presumably if there are odd situations in which multiple equilibrium exist, then the one that we will converge to will have the lowest value of colonization proportion on discharge. Then our A_i values would be an underestimate, and so the qualitative nature of our results will hold.

A_i can be compactly expressed in terms of equilibrium values, solving for the equilibrium values of the compartments. However, solving for these equilibrium values is not so trivial because there are some non-linear terms in our system of eight equations; moreover, the disease-free state is not attainable in the presence of ongoing importation. Thus, we find it more pragmatic to arrive at the value for A_i via simulation.

We have added text about how we set the initial conditions. For fear of distracting the reader with too many technical details, we haven't elaborated further on this issue in the main text. But we welcome input or suggestions for how to make our methods more clear.

> Line 297: These derivatives were evaluated at the disease-free equilibrium, where all infectious compartments are set to zero and susceptible patients are held at their equilibrium value (S = S').

As for a couple of non-technical suggestions that I think would improve the paper: 1. I'd be very interested to see a scatter graph of R_i against A_i to see how strongly correlated they are.

We added a scatter graph as a supplementary figure. We found a positive correlation in outcomes. We added the figure as supplementary material.

> Line 375: We observed a positive correlation in R_i and A_i (S1 Figure).

2. On that note, some discussion of how the amplification is occuring while R_i is less than 1 around 405 would be useful to help explain the concept of what's going on. I'm assuming that it's that while less than

1 person is getting infected on average, the initial person remains infected, so you end up on average with more infections leaving than entered. Is that what's happening?

Yes, we believe this is the main reason. While the intrinsic reproduction number (R_i) is below one, the assumption, supported by previous studies, that most colonized patients remain colonized until discharge can explain the cumulative amplification of colonization. We have clarified this mechanism in the revised section.

> Line 444: By introducing A_i, our analysis indicates a consistent amplification of colonized cases within the healthcare setting, even in scenarios where R_i remains low. This pattern is explained by the persistence of colonization among asymptomatic patients until discharge.

Minor pdf-conversion(?) notes

211/207 The parameter multiplying the E compartment isn't rendering
247/248 Any and all symbols to the left of (x) on these lines aren't rendering

Thanks for noticing this. It is rendering ok for us. We'll keep an eye on the final version to make sure it looks ok

Reviewer #3: This is an infectious disease modelling study that examined the role of healthcare associated C. difficile infection in association with the broader community to solve the 'epidemiological paradox'. The paper is well written and clear. The description of method is clear and in sufficient detail for replication. The inclusion of several sensitivity analyses are useful in understand how the model output could change by different assumptions / definitions. I only have several minor comments:

Line 100: missing '2)' before Suspectable (S)...

Thanks for noticing this. Now corrected.

Lines 205-212: The use of squared brackets confuses the with in-text citation of references.

Thanks for pointing this out. We changed to parenthesis

Lines 284-289: While PRCC it is a standard method in global sensitivity analysis, it does not capture any non-monotonic effect of parameters on outputs. Could the author discuss if effect non-monotonic is possible?

We thank the reviewer for this comment. We conducted univariate sensitivity analyses across the parameter space to assess potential non-monotonic effects on the reproduction number and amplification index. No non-monotonic behavior was observed, and the results are reported in Table S3.

> Lines 393: Within the explored parameter space, we did not observe non-monotonic effects on outcomes (S4 Table).
