# Supplementary material for: Clostridioides difficile colonization amplification despite limited in-hospital transmission: A modeling study
Source: PLoS Med. 2026 Apr 13;23(4):e1004712. doi: 10.1371/journal.pmed.1004712 (PMC13120704; doi:10.1371/journal.pmed.1004712)
Supplement: Attachment — Submitted filename: Responses_auresp_3.pdf [file pmed.1004712.s008.pdf]

Dear Editorial Team,

We thank you for the review of our manuscript and for the helpful comments. We have revised the manuscript accordingly and provide detailed responses to each request below. Changes have been incorporated into the revised manuscript where appropriate.

Sincerely,

Daniel De-la-Rosa-Martínez, MD, PhD
University of California, San Francisco
Institutional email: daniel.delarosamartinez@ucsf.edu
Personal email: dan.delarosam@gmail.com

Seth Blumberg, MD, PhD
University of California, San Francisco
Institutional email: Seth.Blumberg@ucsf.edu

Requests from Editors:

Title needs to adhere to PLOS Medicine criteria. Please confirm that your title complies with PLOS Medicine's style. Your title must be nondeclarative and not a question. It should begin with main concept if possible. "Effect of" should be used only if causality can be inferred, i.e., for an RCT. Please place the study design ("A randomized controlled trial," "A retrospective study," "A modelling study," etc.) in the subtitle (ie, after a colon). *C. difficile* should be spelled out in full at first use in title, line 26 of Abstract, in the Author Summary, and in Introduction.

Response: We have revised the title. The study design has been moved to the subtitle following a colon, and *Clostridioides difficile* is spelled out in full at first use.

Please confirm that all numbers presented in the abstract are present and identical to numbers presented in the main manuscript text.

Response: We confirm that all numerical values reported in the Abstract are identical to those presented in the main manuscript text. We have added to the Results section the numerical values from a sensitivity analysis that was previously shown only in Figure 3 and not reported in the text. The following sentence was added to the manuscript:

> Line 411: Compared with the baseline definition of HCA-CDI (> 3 days post-admission), A_i and R_i increased by 11% and 21%, respectively, when a threshold of ≥ 1 day post-admission was used to define HCA-CDI. Conversely, using a threshold of ≥ 5 days post-admission to

define hospital-acquired disease decreased the baseline values by 5% and 8%, respectively (Fig 3).

Use commas for ranges, not hyphens. Line 37 'Ai'—i should be subscripted. Line 41: CDI needs to be defined at first use.

Response: Commas are now used for ranges, the "i" in Ai has been formatted as a subscript, and *Clostridioides difficile infection (CDI)* is now defined at first use.

Line 49: "which contributes to'—please temper claim as community spread is not directly addressed in this study.

Response: We have tempered the statement to avoid implying direct evidence of community transmission. The sentence now reads:

> Line 52: Our findings indicate that in most potential scenarios, in-hospital transmission of *C. difficile* is limited ($R_i < 1$) and likely sustained by continuous importation of cases from the community. Nevertheless, hospitalization amplifies colonization ($A_i > 1$), which potentially contributes to community transmission.

In the last sentence of the Abstract Methods and Findings section, please describe the main limitation(s) of the study's methodology.

Response: We have added a sentence describing the main methodological limitation of the study at the end of the "Methods and Findings" section of the Abstract:

> Line 48: Our main limitation stems from uncertainty in some parameters describing the disease's natural history, particularly colonization prevalence at admission, progression to symptomatic disease, and the incubation period, which may affect the precision of our estimates despite sensitivity analyses.

Please ensure that the Abstract provides all the information relevant to this study type https://journals.plos.org/plosmedicine/s/submission-guidelines#loc-abstract

Response: Our abstract aligns with PLOS Medicine guidelines.

Line 60: change 'diseased' to 'disease'

Response: This has been corrected to "disease".

In the Author Summary, please limit each section to ~3 bullet points and re-write in an accessible, non-technical manner for a wide audience that includes both scientists and non-scientists. Please include the main limitations of the study in non-technical language in the last bullet point of "What do these findings mean?"

Response: We revised the Author Summary to limit each section. We also included the main study limitation in the final bullet point of the "What Do These Findings Mean?" section.

Please ensure that the Introduction ends with a clear description of the study question or hypothesis.

Response: We have revised the final paragraph of the Introduction:

> Line 117: Our observation that the community-healthcare interface heavily influences the burden of HCA-CDI motivates alternative approaches to assessing within-hospital transmission and evaluating control strategies focused on the importation of asymptomatic carriers of *C. difficile.* In this study, we develop a mathematical model to estimate hospital transmission and assess the sensitivity of transmission metrics to case definition thresholds. We also evaluate the potential impact of interventions for asymptomatic carriers at admission, including screening and contact precautions and/or prophylactic decolonization. Our focus on interventions addressing asymptomatic transmission fills an important research gap, as current infection prevention strategies focus almost exclusively on symptomatic cases, despite substantial evidence that asymptomatic individuals can act as reservoirs for onward transmission in healthcare facilities and the community [12–16].

Please define all abbreviations in Tables and Figures. Please ensure that all Figures and Tables have titles and legends.

Response: Abbreviations have been defined. Figures and Tables include titles and legends.

Please define all elements of box plots in the figure caption - center line, box limits and whiskers.

Response: We added information regarding elements of the box plots.

Please include a Statistical Analysis subsection in the Methods.

Response: “Statistical Analysis” subsection has been identified in the Methods.

Please clarify in the Methods where the data come from and clarify to editors why ethics approval was required.

Response: We clarified this point in the Methods section. The following sentence was added:

> Line 255: Due to the absence of identifiable patient information and use of retrospective data only, our institutional review board declared our study exempt from full review.

If individuals provided feedback on the manuscript, please include them in the Acknowledgments, otherwise remove the section if not persons are to be acknowledged.

Response: The Acknowledgments section has been removed.

Please address the following points derived from Geoffrey P Garnett, Simon Cousens, Timothy B Hallett, Richard Steketee, Neff Walker. Mathematical models in the evaluation of health programmes. (2011) Lancet DOI:10.1016/S0140-6736(10)61505-X:

Please provide a diagram that shows the model structure, including how the disease natural history is represented, the process and determinants of disease acquisition, and how the putative intervention could affect the system.

Response: A schematic diagram illustrating the compartmental model structure, including disease natural history, acquisition processes, and the mechanisms through which interventions act, is provided in Figure 1 and described in the Methods (Model structure) section.

Please provide a complete list of model parameters, including clear and precise descriptions of the meaning of each parameter, together with the values or ranges for each, with justification or the primary source cited, and important caveats about the use of these values noted.

Response: A complete list of parameters, including their definitions, baseline values, ranges explored, and sources, is presented in Table 1. In addition, model structure is described in detail in the model description section.

Please provide a clear statement about how the model was fitted to the data including where relevant goodness-of-fit measure, the numerical algorithm used, which parameter varied, constraints imposed on parameter values, and starting conditions.

Response: The model calibration procedure is described in the Methods section:

> Line 258: We implemented our compartmental model using the deSolve package in R (version 4.3.2). To incorporate uncertainty in parameter values, we conducted 1,000 simulations in which model parameters, except for the transmission rate, were independently sampled from uniform distributions within ±20% of their baseline estimates (Table 1). The transmission rate or δ was optimized in each simulation through numerical calibration by minimizing the absolute difference between the model-predicted cumulative incidence of CDI, quantified as the total number of individuals passing through compartment A, and the observed number of infections over the study period. Initial conditions for simulations were set at the disease-free equilibrium.

For uncertainty analyses, please state the sources of uncertainties quantified and not quantified this can include parameter, data, and model structure.

Response: Parameter uncertainty was quantified through parameter sampling and sensitivity analyses. We also clarified in the manuscript that uncertainty arising from model structure and between-population heterogeneity was not quantified (line 530).

Please provide sensitivity analyses to identify which parameter values are most important in the model. Uncertainty estimates seek to derive a range of credible results on the basis of an exploration of the range of reasonable parameter values. The choice of method should be presented and justified.

Response: Global sensitivity analyses using partial rank correlation coefficients (PRCC) and univariate sensitivity analyses were performed to identify parameters most influential for the outcomes of interest (Line 324).

Please discuss the scientific rationale for this choice of model structure and identify points where this choice could influence conclusions drawn. Please also describe the strength of the scientific basis underlying the key model assumptions.

Response: The rationale for the chosen model structure and key assumptions is discussed in the model description and discussion, including potential implications of model simplifications such as homogeneous mixing and uncertainties related to poorly characterized aspects of the disease's natural history.
