# Supplementary material for: Clostridioides difficile colonization amplification despite limited in-hospital transmission: A modeling study
Source: PLoS Med. 2026 Apr 13;23(4):e1004712. doi: 10.1371/journal.pmed.1004712 (PMC13120704; doi:10.1371/journal.pmed.1004712)
Supplement: Attachment — Submitted filename: Response.docx [file pmed.1004712.s006.docx]

**Response to Editorial Request:**

We thank the editors for their thoughtful comments and for the opportunity to revise our manuscript. Below, we provide a point-by-point response to each request. The revised manuscript has been prepared and submitted in accordance with the PLOS Medicine guidelines.

We believe these changes have strengthened the clarity and contribution of our work.

Sincerely,

**Daniel De-la-Rosa-Martínez, MD, PhD**

University of California, San Francisco

**Institutional email**: [daniel.delarosamartinez@ucsf.edu](mailto:daniel.delarosamartinez@ucsf.edu)

**Personal email**: [dan.delarosam@gmail.com](mailto:dan.delarosam@gmail.com)

**Revision of Title and Abstract**

We have revised both the title and the abstract to align with PLOS Medicine specifications.

**Data Availability Statement and Code Deposition in Public Repository:**

We included a data availability statement clarifying that no individual-level patient data were used. All code and data necessary to reproduce the analyses are openly available at the following GitHub repository: <https://github.com/danieldelarosam/C-difficile-Transmission-Model.git>

**Acknowledgments Section:**

We confirm there are no acknowledgments to declare (Page 21, line 470)

**Ethics Statement:**

We clarified the Ethics Statement in the manuscript during submission. This study did not involve human participants, interventions, or identifiable data. Only aggregate, de-identified census data from the UCSF Medical Center were used. No ethics approval was required.

**Identification of Data Source Institution:**

We explicitly identified UCSF Medical Center as the data source in the Methods section (page 9, lines 215–218).

**Discussion of Study Limitations and Generalizability:**

We have expanded the Discussion section to more explicitly address the limitations of our work, particularly concerning generalizability. As we now note, specific estimates of Ri and Ai may vary depending on local factors such as demographics, infection control practices, and antibiotic use. However, the fundamental epidemiological processes captured by our model are generalizable and adaptable to other settings.

**Page 20, Line 462:** Lastly, the generalizability of our findings should be considered with caution, as specific estimates of R_i_ and A_i_ may vary across institutions depending on local patient demographics, sources of admission, infection control practices, and patterns of antibiotic use. Our model structure captures fundamental and generalizable epidemiological processes **underlying** *C. difficile* transmission that are applicable to other healthcare settings and can be flexibly adapted with a limited set of parameters. While the aforementioned limitations inspire opportunities for future work, our model provides insight for assessing the transmission dynamics and control of *C. difficile* in healthcare settings.

**Policy or Clinical Impact and Advancement Over Prior Studies:**

We expanded the Discussion to clarify the policy and clinical implications of our findings. Unlike prior studies, our model explicitly quantifies colonization amplification and interventions targeting asymptomatic carriers.

**Page 18, Line 409:** Unlike previous studies, our model explicitly quantifies colonization amplification and incorporates interventions targeting asymptomatic carriers. This underscores the need to expand strategies beyond managing symptomatic cases to include interventions targeting asymptomatic carriers at the time of admission **and, potentially,** at discharge. From a policy perspective, our results suggest that relying solely on R_i_ to evaluate hospital performance may underestimate the broader epidemiological impact of healthcare institutions on *C. difficile* transmission.

**Page 19, Line 431:** Although quasi-experimental studies have supported the effectiveness of contact precautions [30],  strategies to prevent the progression from asymptomatic carriage to active infection may be particularly valuable in settings where transmission from asymptomatic carriers is not the primary driver of spread. For example, Miles-Jay et al. showed that in intensive care units (ICUs), only 1% of patients who tested negative for *C. difficile* upon admission acquired the pathogen through cross-transmission, with most cases occurring in previously colonized patients. However, it is essential to note that conditions in ICUs may not reflect those in general hospital wards. **ICU patients were typically housed in single rooms and benefited from daily cleaning with sporicidal disinfectants, which likely reduced opportunities for cross-transmission compared to other healthcare settings** [31].

**Divergent views in terms of how healthcare-associated CDIs are sustained:**

Our findings contribute to the ongoing gap regarding the driver of healthcare-associated CDI supporting the role of both importation and within-hospital amplification dynamics:

**Page 18, Line 394:** Our findings highlight the bidirectional relationship between hospital and community *C. difficile* burden. Based on our assumptions, the consistently low R_i_ (<1) across scenarios suggests that in-hospital transmission alone does not sustain endemicity. Instead, ongoing importation of cases from the community is necessary to maintain endemicity within healthcare institutions. This highlights the importance of accounting for internal and external dynamics in infection control strategies [26,27].

**Page 18, Line 400:** Meanwhile, hospitals contribute to the broader community burden by discharging colonized individuals, many of whom remain undetected by current screening practices. By introducing the A_i_, our analysis indicates a consistent amplification of colonized cases within the healthcare setting, even in scenarios where R_i_ remains low. These findings align with and complement previous reports documenting increased colonization at discharge, higher risk of CDI among individuals recently exposed to healthcare settings, and studies emphasizing the role of community-hospital interactions [26,28,29]. This hospital-driven amplification may indirectly sustain healthcare-associated transmission if colonized individuals are later readmitted.
